# First detection of West Nile virus in Belgium through wild bird surveillance, Belgium, 2025

**DOI:** 10.2807/1560-7917.ES.2026.31.4.2600049

**Published:** 2026-01-29

**Authors:** Charlotte Sohier, Floris C Breman, Muriel Vervaeke, Reina Sikkema, Marjan Boter, Bas Oude Munnink, Marion Koopmans, Annick Linden, Laura Duran Illan, Javiera Rebolledo Romero, Tinne Lernout, Nick De Regge

**Affiliations:** 1 Sciensano, Belgian Health Institute, Brussels, Belgium; 2 Agency for Nature and Forests, Brussels, Belgium; 3 Erasmus MC, Rotterdam, the Netherlands; 4 FARAH, Faculty of Veterinary Medicine, University of Liège, Liège, Belgium; Correspondence: Charlotte Sohier (Charlotte.Sohier@sciensano.be)

**Keywords:** West Nile virus, Belgium, wildlife surveillance, One Health, lineage 2

## Abstract

In August 2025, West Nile virus (WNV) was detected for the first time in Belgium through a monitoring programme in wild birds, with three corvids testing positive by RT-qPCR. In September and October, four additional infected birds were identified. Whole genome sequencing classified the strain as WNV lineage 2, consistent with strains circulating elsewhere in Europe. These detections provide evidence of local WNV circulation with important implications for animal and public health preparedness and surveillance during the 2026 mosquito season.

Following the first identification of West Nile virus (WNV) in wild birds in Belgium in August 2025, genomic characterisation was performed to place the finding in its appropriate epidemiological context. Over the past decade, WNV has expanded its geographical range across Europe, with sustained transmission reported in several countries bordering Belgium, including France, Germany and the Netherlands [1-4]. This rapid communication documents this detection event in Belgium and outlines its relevance for public and animal health.

## Detection of West Nile virus in wild birds

*As part of Belgium’s national One health for surveillance (OH4S) programme* for WNV*,* wild birds admitted to participating rehabilitation centres between May and October—typically due to injury or poor condition—are sampled using oropharyngeal swabs and screened for West Nile virus and Usutu virus (USUV). The timing of sample testing differed between centres, as most stored all collected samples over the surveillance period and tested them later in batches. Samples collected at the Malderen wildlife rehabilitation centre were submitted in three batches to Sciensano in July, September and November 2025.

**Figure 1:**
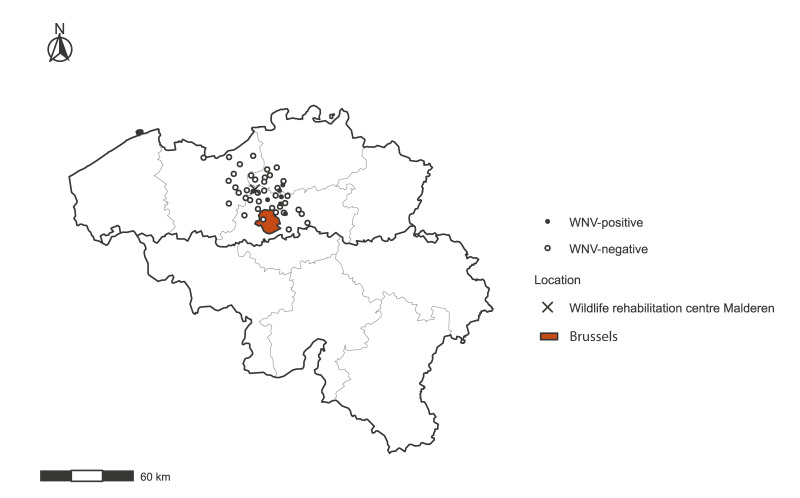
Geographical origin of sampled wild birds admitted to the Malderen wildlife rehabilitation centre and tested for West Nile virus, Belgium, May–October 2025 (n = 113)

The wildlife rehabilitation centre in Malderen collected 113 samples between May and October 2025. They were tested for WNV RNA by real-time reverse transcription PCR (RT-PCR) using previously described assays [5-8]. Seven wild birds swabbed between 2 August and 21 October tested qRT-PCR-positive for WNV. The first detections occurred in swabs collected in August and involved one carrion crow (*Corvus corone*) and two Eurasian jackdaws (*Corvus monedula*) from Meise, Mechelen and Hombeek. All three birds were admitted to the wildlife rehabilitation centre in poor condition (trauma or exhaustion) and died shortly after admission. Their quantification cycle (Cq) values ranged from 28.2 to 32.8. A second cluster of detections was made in swabs collected in September and October and included one northern goshawk (*Accipiter gentilis*), one carrion crow and two Eurasian jackdaws originating from Buggenhout, Zaventem, Vilvoorde and Zemst. These birds were brought to the rehabilitation centre due to exhaustion, collision injuries or entrapment, and showed Cq values between 32.4 and 35.3. Of these four birds, the carrion crow from Vilvoorde and the Eurasian jackdaw from Zaventem died shortly after admission, whereas the absence of further information on the northern goshawk from Buggenhout and the Eurasian jackdaw from Zemst suggests that these survived and were released. Figure 1 provides a map of the geographical origin of birds that were admitted to the Malderen wildlife rehabilitation centre which were swabbed and tested for WNV. All WNV-positive samples tested negative for USUV using specific RT-PCR assays.

Map of Belgium. Black dots represent WNV-positive birds; open circles represent WNV-negative birds. The location of the Malderen wildlife rehabilitation centre is indicated by a cross. A total of 113 birds were sampled; overlapping points occur due to multiple birds originating from the same or nearby locations.

## Genomic characterisation

**Figure 2:**
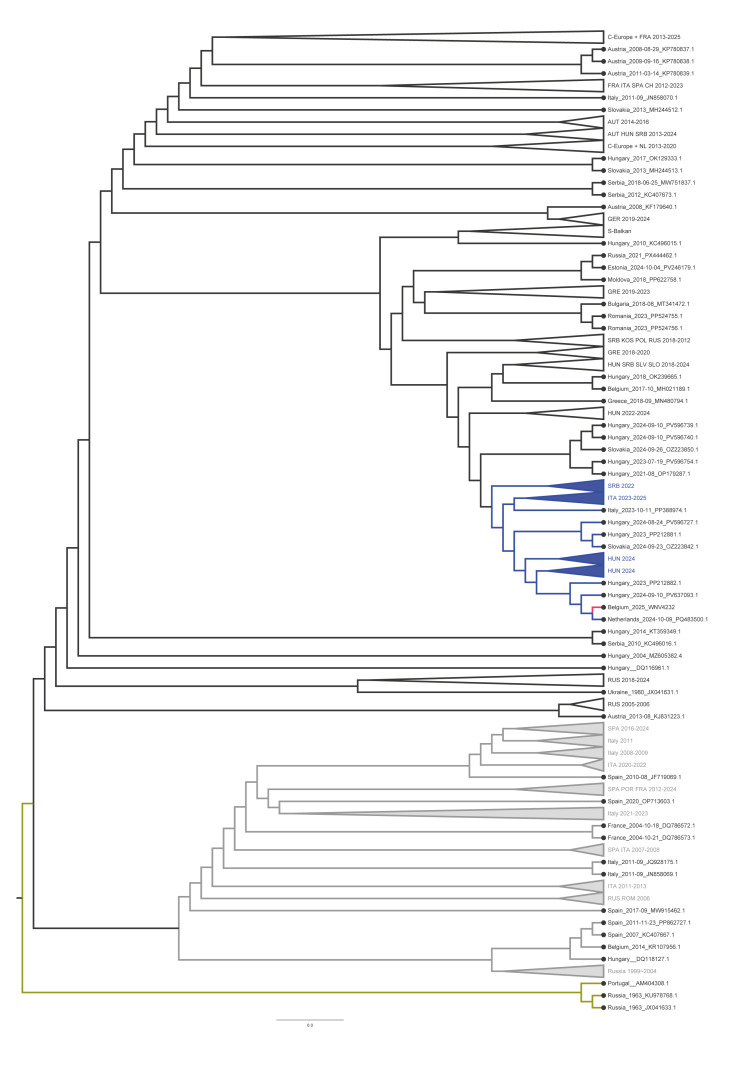
Maximum-likelihood phylogeny of the complete coding sequence of a West Nile virus strain isolated from a carrion crow, Belgium, May–October 2025 (n = 1) compared with available European West Nile virus sequences

We performed amplicon-based whole genome sequencing with the methodology described in Sikkema et al. [9]. The swab from the carrion crow from the first batch, which had the lowest Cq value (28.23), yielded RNA of sufficient quality to allow whole genome determination. We performed phylogenetic analysis using a maximum-likelihood approach as previously described [10] and show that the sequence clustered within a lineage 2 clade that has been circulating widely in central and western Europe since ca 2010 [11]. The Belgian sequence grouped closely with recent sequences detected in Hungary during 2023 and 2024 and with a 2024 sequence from the Netherlands that was obtained from a human infection in a traveller returning from Spain (Figure 2). The Belgian WNV genome sequence from 2025 has been deposited in GenBank (accession number: PX906586). The phylogeny also includes two non-autochthonous WNV sequences previously deposited in GenBank under a Belgian country label (MH0221189.1 and KR107956.1). The MH0221189.1 sequence corresponds to a virus detected in a traveller returning from Hungary, while KR107956.1 represents a laboratory clone of the Israeli strain Is98. Neither sequence reflects prior autochthonous WNV circulation in Belgium.

AUT: Austria; BEL: Belgium; BUL: Bulgaria; CH: Switzerland; CZ: Czech Republic; EST: Estonia; FRA: France; GER: Germany; GRE: Greece; HUN: Hungary; KOS: Kosovo‡; NL: the Netherlands; POL: Poland; POR: Portugal; ROM: Romania; RUS: Russia; SLO: Slovenia; SLV: Slovakia; SPA: Spain; SRB: Serbia; UKR: Ukraine.

Lineage 1 sequences are greyed out, and the oldest known WNV-1 lineages are shown in green. Lineage 2 strains are shown in black. The clade containing the Belgian strain from 2025 is highlighted in blue, with the Belgian strain and corresponding branch highlighted in red. Node support was assessed using Shimodaira–Hasegawa approximate likelihood ratio test (SH-aLRT), approximate Bayes test and ultrafast bootstrap; supported clades are indicated by an asterisk.

## Animal and public health actions

Following the first positive laboratory result, reported on 1 October 2025 for samples that had been collected in August, the Belgian federal and regional authorities were informed, and reported the detections to the Animal Disease Information System (ADIS) and the World Organisation for Animal Health (WOAH), in accordance with international reporting requirements. The findings prompted the convening of the Risk Assessment Group on Veterinary Emerging Zoonoses (RAG-V-EZ), which recommended further monitoring of virus circulation in birds without recommending additional control measures in domestic animals at this stage. Preventive recommendations were, however, provided to wildlife rehabilitation centres, including advice on reducing exposure of staff and birds to mosquito bites through personal protective measures and improved mosquito-proofing of indoor enclosures. As there have been WNV cases in neighbouring countries, the human Risk Assessment Group (RAG) considered these findings not unexpected and assessed the risk of additional transmission as low as the notification occurred towards the end of the vector season.

Information on the detections was published by the competent authorities for animal health, human health and wildlife, and communicated through the national Newsflash. The regional health authority informed neurologists in Flanders by letter and asked them to reinforce clinical awareness during the next mosquito season. As indicated above, preventive recommendations were also provided to wildlife rehabilitation centres. The Federal Agency for the Safety of the Food Chain informed veterinarians to raise awareness of possible equine cases.

## Discussion

The detection of WNV in several wild birds over a period of 3 months is evidence of local WNV circulation in Belgium and constitutes an early warning signal for potential spillover to humans and horses. Strain identification showed that the detected virus belongs to lineage 2, one of the main lineages circulating in Europe in both avian and human populations, highlighting the public health relevance of these detections.

*These findings should be interpreted within the broader European epidemiological context.* Although Belgium has long been considered at risk because of the widespread presence of competent *Culex* mosquito vectors, it was one of the few European countries with no reports of autochthonous WNV infections in humans and animals [12,13]. This absence of detected circulation was probably influenced, at least in part, by the limited surveillance activities in place, which relied primarily on passive surveillance in humans (since 2012) and in dead wild birds (starting in 2010 in Flanders and in 2017 in Wallonia [14]). In addition, in 2022 and 2023, limited active WNV monitoring was carried out in Flanders, where oral swabs were collected from wild birds submitted to two wildlife rehabilitation centres [10]. 

*To address these gaps, active monitoring was strengthened nationwide.* Since 2024, active WNV monitoring has been intensified and carried out in 15 wildlife rehabilitation centres throughout Belgium as part of the OH4S project, a European initiative aimed at strengthening early detection of emerging and re-emerging zoonotic pathogens through coordinated surveillance in animals, vectors and the environment [15]. In Belgium, OH4S West Nile virus surveillance is implemented by a collaborative initiative between Sciensano, the University of Liège and the regional authorities for wildlife of Flanders (Agency for Nature and Forests), Wallonia (Walloon Public Service) and Brussels (Brussels Environment).

These detections, which were identified through active surveillance, illustrate the added value of this approach for the early detection of WNV circulation in Belgium. However, passive surveillance of dead wild birds remains a highly sensitive and cost-effective tool for early detection of WNV circulation, but the number of submitted samples in Belgium remains limited and geographically uneven. The birds found WNV-positive in August died shortly after admission and would therefore probably have been identified through passive dead-bird surveillance, **if** they had been reported as carcasses. Active sampling in rehabilitation centres complements this passive surveillance system by enabling systematic sampling across all provinces, detecting infections in birds that survive a WNV infection and would not enter passive surveillance. The active wild bird surveillance therefore contributes to early detection in situations where carcass reporting or recovery is limited.

After action reviews carried out in several European countries (Greece, Italy, Serbia, Slovenia) in response to the severe 2018 West Nile virus transmission season concluded that coordinated, cross-sectoral One Health preparedness is essential for managing such events [16]. In these reviews, all four participating countries indicated that having a One Health surveillance and response plan was critical in the management of the event. Although Belgium currently does not have a WNV preparedness plan, this first confirmation of WNV circulation was an important wake-up call for the Belgian authorities. In 2026, a national preparedness plan will be further elaborated, which should identify gaps and needs for enhanced surveillance and enable appropriate intervention measures depending on the level of virus circulation, including measures for blood safety and vector control as well as communication plans.

Furthermore, wild bird samples collected at the other rehabilitation centres within the OH4S project are being tested to obtain a more complete overview of WNV and USUV circulation in Belgium during the 2025 transmission season. An increased frequency of sample testing is planned for 2026 to support more timely detection and improved situational awareness. 

## Conclusion

The first detection of WNV circulation in wild birds in Belgium marks an important early warning signal for potential spillover to humans and horses. Genomic characterisation confirmed that the detected virus belongs to lineage 2, consistent with strains circulating elsewhere in Europe. These findings highlight the need for continued integrated One Health surveillance to ensure timely detection of virus circulation and to support early public and animal health preparedness.

## Data Availability

The Belgian WNV whole genome sequence has been submitted to GenBank, accession number PX906586.

## ^‡^Note

This designation is without prejudice to positions on status, and is in line with UNSCR 1244/99 and the IСJ Opinion on the Kosovo declaration of independence.
